# Pharmacogenomics in Psychiatry: An Asian Perspective

**DOI:** 10.1192/j.eurpsy.2024.694

**Published:** 2024-08-27

**Authors:** S. E. Goh

**Affiliations:** Institute of Mental Health, Singapore, Singapore

## Abstract

**Introduction:**

Pharmacogenomic testing in psychiatry is an emerging area with the potential clinical application of guiding medication choice and dosing. However, this has not been adopted widely due to a combination of barriers that include a varying evidence base, clinician and patient familiarity and acceptance, uncertainty about cost-effectiveness, and regulatory requirements.

**Objectives:**

This review aims to examine recent updates in this field and provide a contextualised summary and recommendations for Asian populations. The recommendations serve to guide healthcare professionals in the utility of pharmacogenomic testing in psychiatric practice.

**Methods:**

A review of recent literature about current evidence and guidelines surrounding pharmacogenomics in psychiatric practice was carried out with particular attention paid to literature evaluating Asian populations. Literature was reviewed for the different classes of psychotropics with supplementary information about Asian populations included where available. Existing evidence about combinatorial pharmacogenomic panels was also reviewed.

**Results:**

In line with the available body of evidence, we recommend that pharmacogenomic testing should be employed as an augmenting tool to guide medication selection and dosing in certain clinical situations, and not as part of standard or routine clinical practice. Pharmacogenomic testing should also be mainly limited to the known drug-gene pairs such as the anti-depressants and CYP2C19 or CYP2D6. Clinicians should also be aware that many of the gene-drug associations have not been evaluated for clinical outcomes. Combinatorial pharmacogenomic panels are not presently recommended as there is limited and inconclusive available evidence on clinical outcomes.

**Conclusions:**

Pharmacogenomic testing in psychiatry is not recommended as standard or routine clinical practice. Exceptions may include concerns about drug concentrations (due to metaboliser status) or potential severe adverse drug reactions/ Pharmacogenomic testing should be mainly limited to the known drug-gene pairs such as the anti-depressants and *CYP2C19* or *CYP2D6.*

**Disclosure of Interest:**

None Declared

